# Attraction of *Halyomorpha halys* (Hemiptera: Pentatomidae) haplotypes in North America and Europe to baited traps

**DOI:** 10.1038/s41598-017-17233-0

**Published:** 2017-12-05

**Authors:** William R. Morrison, Panos Milonas, Despoina Evr. Kapantaidaki, Michele Cesari, Emanuele Di Bella, Roberto Guidetti, Tim Haye, Lara Maistrello, Silvia T. Moraglio, Lucia Piemontese, Alberto Pozzebon, Giulia Ruocco, Brent D. Short, Luciana Tavella, Gábor Vétek, Tracy C. Leskey

**Affiliations:** 1 USDA, Agricultural Research Service, Center for Grain and Animal Health Research, 1515 College Ave., Manhattan, KS 66502 USA; 2 Department of Entomology and Agricultural Zoology, Benaki Phytopathological Institute, 8 St. Delta str., Kifissia, Greece; 3 Department of Life Sciences, University of Modena and Reggio Emilia, Via G. Amendola 2, Reggio Emilia, and via Campi 213/D, Modena, Italy; 4 CABI, Rue des Grillons 1, 2800 Delémont, Switzerland; 5 Dipartimento di Scienze Agrarie, Forestali e Alimentari, University of Turin, Largo P. Braccini 2, 10095 Grugliasco, TO Italy; 6 Department of Agronomy, Food, Natural Resources, Animals and Environment – University of Padova, viale dell’Università, 16, 35020 Legnaro, PD Italy; 7 USDA, Agricultural Research Service, Appalachian Fruit Research Station, 2217 Wiltshire Rd., Kearneysville, WV 25430 USA; 8 Department of Entomology, Szent István University, Villányi út 29–43, H-1118 Budapest, Hungary

## Abstract

*Halyomorpha halys* is a global invasive species, native to Southeast Asia, that is threatening agriculture in invaded regions. Our objectives were to: 1) establish the attractiveness of semiochemical stimuli paired with field-deployed traps in Europe (Greece, Hungary, Italy, and Switzerland), compared with Maryland, USA, and 2) identify *H. halys* haplotypes recovered from traps at each location. We found qualitatively identical patterns of capture between sites located across Europe and in Maryland, USA. In both regions, captures of *H. halys* adults indicated a synergistic response to traps baited with the two component *H. halys* aggregation pheromone, and pheromone synergist, methyl (2*E*, 4*E*, 6*Z*)-decatrienoate when compared with either individually. Haplotype diversity in Europe based on trapped specimens was much greater than the USA, with five new haplotypes described here, probably indicating ongoing invasion and re-introduction of *H. halys*. By contrast, a single, previously identified haplotype was trapped in Maryland, USA, representing a single introduction. All *H. halys* haplotypes responded to each semiochemical in apparent proportion to their frequency in the overall population based on independently derived information from prior work. Taken together, these data suggest that pheromone-based technology will be of global utility for the monitoring of this important invasive species.

## Introduction

The brown marmorated stink bug, *Halyomorpha halys* (Stål) (Hemiptera: Pentatomidae), is a global invasive species. It is originally from Southeast Asia, including Japan, the Republic of Korea, and China^1–3^. However, it has expanded its range, first invading the United States^4^ where it caused enormous agricultural damage over the past decade^5,6^, subsequently invading Canada^7^, 12 countries in Europe^8–23^, Russia, Abkhazia, Georgia^24^ and is now established in South America^25^. Niche modeling and other data indicate that its range is only projected to increase further in the coming years^26,27^, and it is a significant biosecurity concern for several countries, including Australia and New Zealand^28–30^.

There has been substantial work on elucidating the invasion pathways for *H. halys* in the countries where it is present based on the sequencing of genetic haplotypes. For example, the population of *H. halys* in the United States likely originated from a single introduction of a small propagule of individuals from Beijing, China^31^. To the north, the populations of *H. halys* in Canada appear to have been derived from the introduction and subsequent movement of conspecifics from the United States^32^. By contrast, Europe has undergone several introductions, including directly from Asia for *H. halys* populations in France and Switzerland, as well as possibly via the United States and through secondary invasions for populations in Italy, Hungary, and Greece^8,33,34^.

In the past decade, there have been marked advances in developing reliable pheromone-based technology for monitoring *H. halys* (reviewed in^35^). Large black pyramid traps were found to be effective at capturing *H. halys* when baited with the cross-attractive *Plautia ståli*-produced aggregation pheromone, methyl (2*E*,4*E*,6*Z*)-2,4,6-decatrienoate^36^ (hereafter, MDT), but only during the late season. Khrimian *et al*.^37^ discovered the *H. halys* male-produced aggregation pheromone as the two components, (3*S*,6*S*,7*R*,10*S*)-10,11-epoxy-1-bisabolen-3-ol and (3*R*,6*S*,7*R*,10*S*)-10,11-epoxy-1-bisabolen-3-ol in a 3.5:1 ratio (hereafter, “PHER”). Extreme purity of PHER is not required, as other stereoisomers are not inhibitory, and some isomers not found naturally may even be attractive^38^. Weber *et al*.^39^ found that MDT synergizes attraction to PHER when deployed in traps. The combination of PHER and MDT provided for reliable season-long attraction of *H. halys* adults and nymphs in North America^40^ as well as Asia^41^. With these effective olfactory stimuli, alternative trap designs also appear sensitive and reliable^42^, including the use of sticky traps (Leskey *et al*. unpublished data). However, pheromone-baited traps are not attractive to *H. halys* during its diapause period^43^, and specific host plant volatiles do not appear to increase attractiveness of existing lures in the field^44^.

Moreover, very little is known about the population-level genetic basis of *H. halys*’ olfactory response. For example, for the mitochondrial cytochrome oxidase I (COI) gene, four, three, and 19 haplotypes have been described from the United States, Canada, and Europe, respectively^8,12,31,33,34,45^, but it is unknown whether all of these haplotypes respond similarly to the combined semiochemicals discussed above. Our objectives were to: 1) establish the attractiveness of semiochemical stimuli paired with field-deployed traps in Europe (Greece, Hungary, Italy, and Switzerland), compared with Maryland, USA, and 2) identify *H. halys* haplotypes recovered from traps at each location.

## Results

### Trapping Study

**Figure 1:**
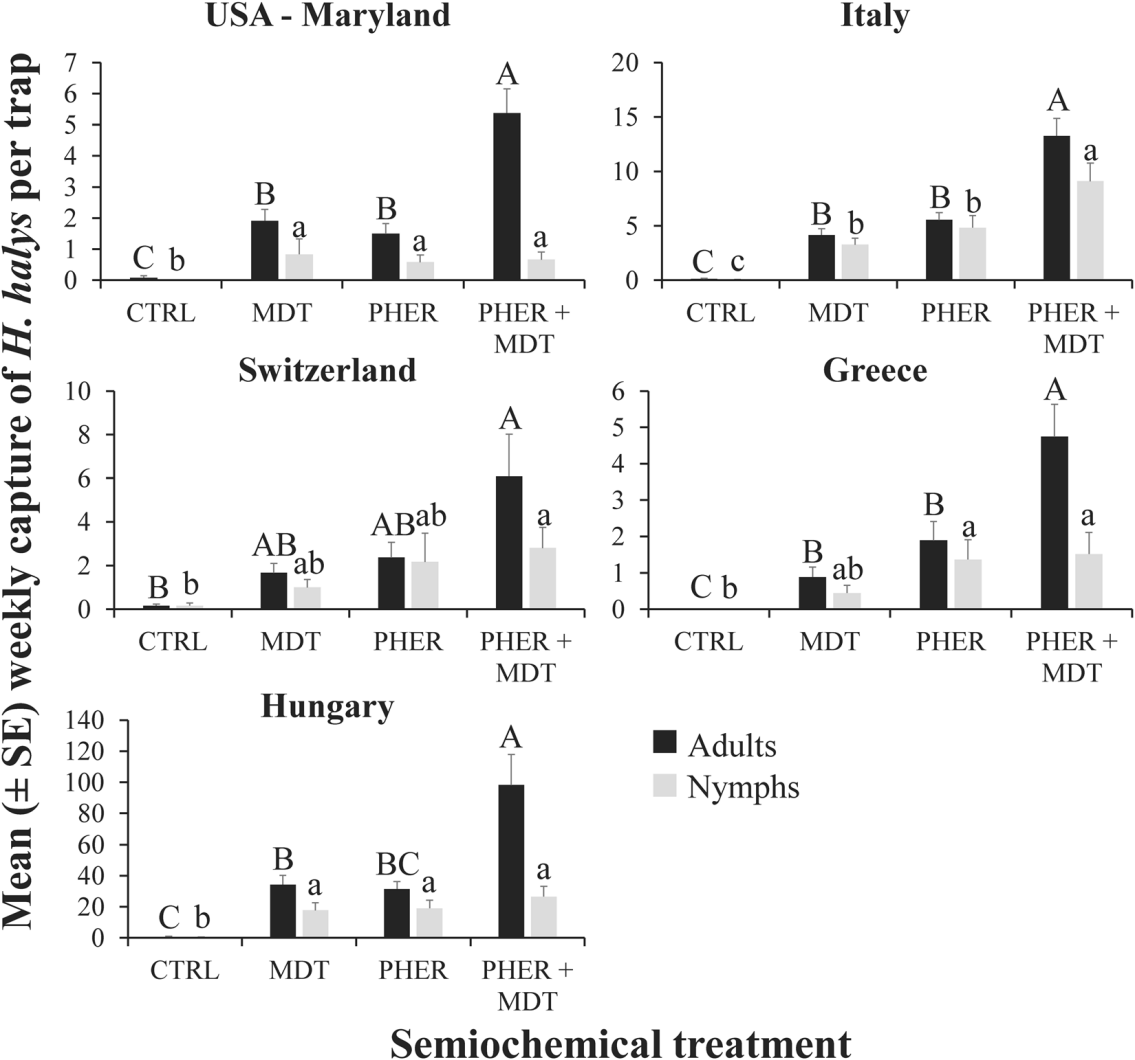
Mean captures of *H. halys* adults (black bars) and nymphs (grey bars) to semiochemical stimuli paired with clear sticky traps by sampled country (Greece, Hungary, Italy, Switzerland, and USA) from 8 Aug to 6 Oct 2016. Upper case letters represent pairwise comparisons among treatments within adults, while lower case letters represent pairwise comparisons within nymphs. Bars with shared letters are not significantly different from each other (Tukey’s HSD, α = 0.05). Abbreviations: PHER = *H. halys* aggregation pheromone, MDT = methyl (2*E*,4*E*,6*Z*)-2,4,6-decatrienoate, CTRL = unbaited control.

**Figure 2:**
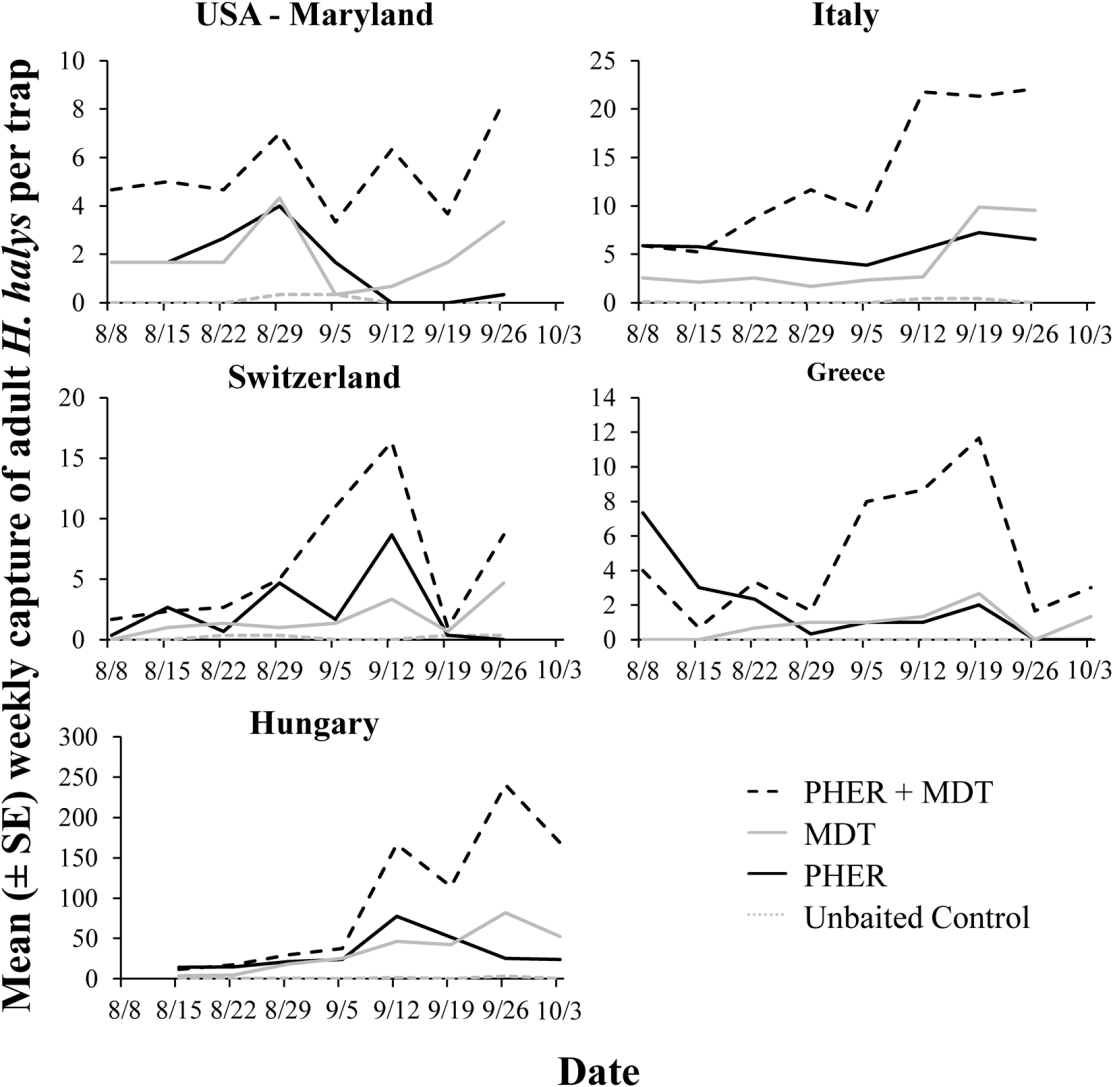
Weekly population dynamics of adult *H. halys*, depending on semiochemical treatment, from 8 Aug to 6 Oct 2016 in each country (Greece, Hungary, Italy, Switzerland, and USA). Abbreviations: PHER = *H. halys* aggregation pheromone, MDT = methyl (2*E*,4*E*,6*Z*)-2,4,6-decatrienoate, CTRL = unbaited control.

In total, 213 and 6,067*H. halys* adults were captured in the USA (Maryland) and Europe, respectively, with 65% of the adults in Europe captured in Hungary. The presence of MDT, PHER, or both when deployed with the sticky cards significantly increased the trap capture of adults (LMM: χ^2^ = 13.0; df = 3; *P* < 0.02; Fig. 1). For example, traps with the combined PHER + MDT in Europe captured over 8 and 2 times more adults than traps that were unbaited, or were baited with PHER or MDT alone, respectively; traps with the PHER + MDT in Maryland captured 65 and 3–4 times more adults compared to traps that were unbaited or only had just one of the stimuli, respectively. The country in which sampling took place (Greece, Hungary, Italy, Switzerland, and USA) significantly affected captures (χ^2^ = 67.32; df = 4; *P* < 0.0001). However, this was primarily driven by the different absolute population pressure in each country, and not the qualitative pattern of capture (notice, for instance, the scales in Fig. 1 which max out at 4 adults for some countries but range up to 70 for others). The sampling date significantly affected trap capture (χ^2^ = 28.4; df = 8; *P* < 0.001), with numerically more adults captured near the end of the sampling period than the beginning (Fig. 2). Traps baited with PHER + MDT captured more adults on every sampling week in each country except for the first two sampling dates when adult populations were low in European countries (Fig. 2). The qualitative patterns in trap capture among treatments were similar between European countries and the USA (Fig. 1). Finally, the presence of MDT with the aggregation pheromone had a synergistic effect on attraction of adults in Greece (*t* = 2.75; df = 27; *P* < 0.01), Hungary (*t* = 3.32; df = 24; *P* < 0.01), Italy (*t* = 4.51; df = 72; *P* < 0.0001), Switzerland (*t* = 2.30; df = 24; *P* < 0.05), and the USA (*t* = 4.59; df = 24; *P* < 0.0001), resulting in between 2.4–3.6 times greater trap capture, respectively, than when traps only had the *H. halys* aggregation pheromone.

**Figure 3:**
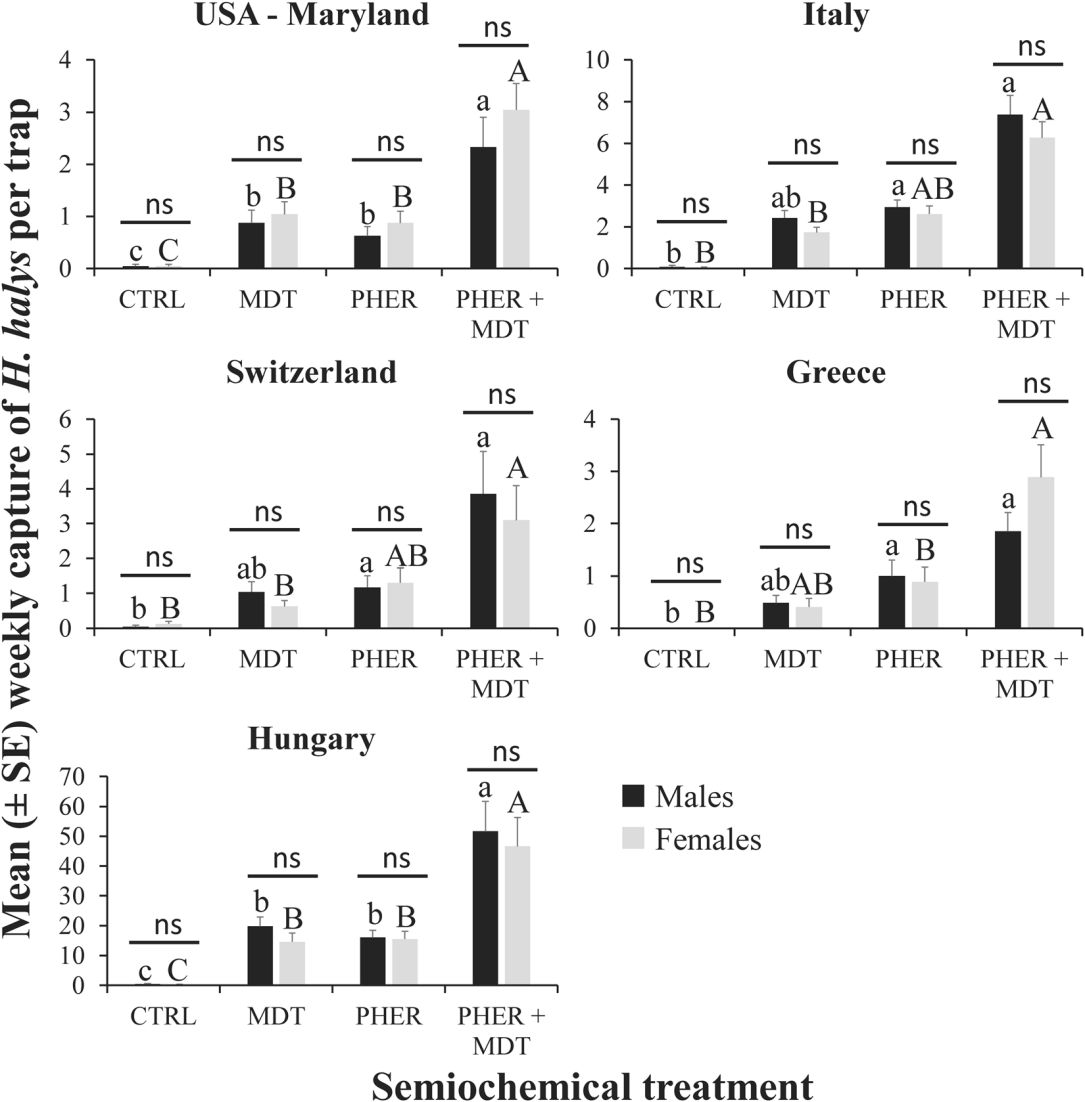
Mean captures of males (black bars) and females (grey bars) by semiochemical stimuli paired with clear sticky traps in each country (Greece, Hungary, Italy, Switzerland, and USA) from 8 Aug to 6 Oct 2016. Upper case letters represent pairwise comparisons among treatments within females, while lower case letters represent pairwise comparisons within males. Bars with shared letters are not significantly different from each other (Tukey’s HSD, α = 0.05). Post-hoc comparisons between male and female response to each treatment are represented by bars, with ns indicating no significant differences (Bonferroni-corrected t-test). Abbreviations: PHER = *H. halys* aggregation pheromone, MDT = methyl (2*E*,4*E*,6*Z*)-2,4,6-decatrienoate, and CTRL = unbaited control.

Moreover, both captures of males (χ^2^ = 25.53; df = 3; *P* < 0.0001) and females (χ^2^ = 27.54; df = 3; *P* < 0.0001) were increased by the addition of MDT, PHER, or both in each country in which traps were deployed (Fig. 3). For both males and females, 8 times more individuals were captured in traps with the PHER + MDT compared to the unbaited control, while over twice as many were captured compared to the traps with PHER or MDT alone. The sampling country significantly affected the abundance of both males (χ^2^ = 38.9; df = 4; *P* < 0.0001) and females (χ^2^ = 61.1; df = 4; *P* < 0.0001). Moreover, the sampling date influenced both the abundance of males (χ^2^ = 34.9; df = 8; *P* < 0.0001) and females (χ^2^ = 67.5; df = 8; *P* < 0.0001), with populations of both peaking near the end of the sampling period. For both males and females, captures on traps with the PHER + MDT were consistently higher than traps with either stimuli alone or that were left unbaited throughout the sampling period. Importantly, there was no sexual dimorphism in response to any of the semiochemical treatments in Europe or Maryland (Fig. 3, t-tests).

**Figure 4:**
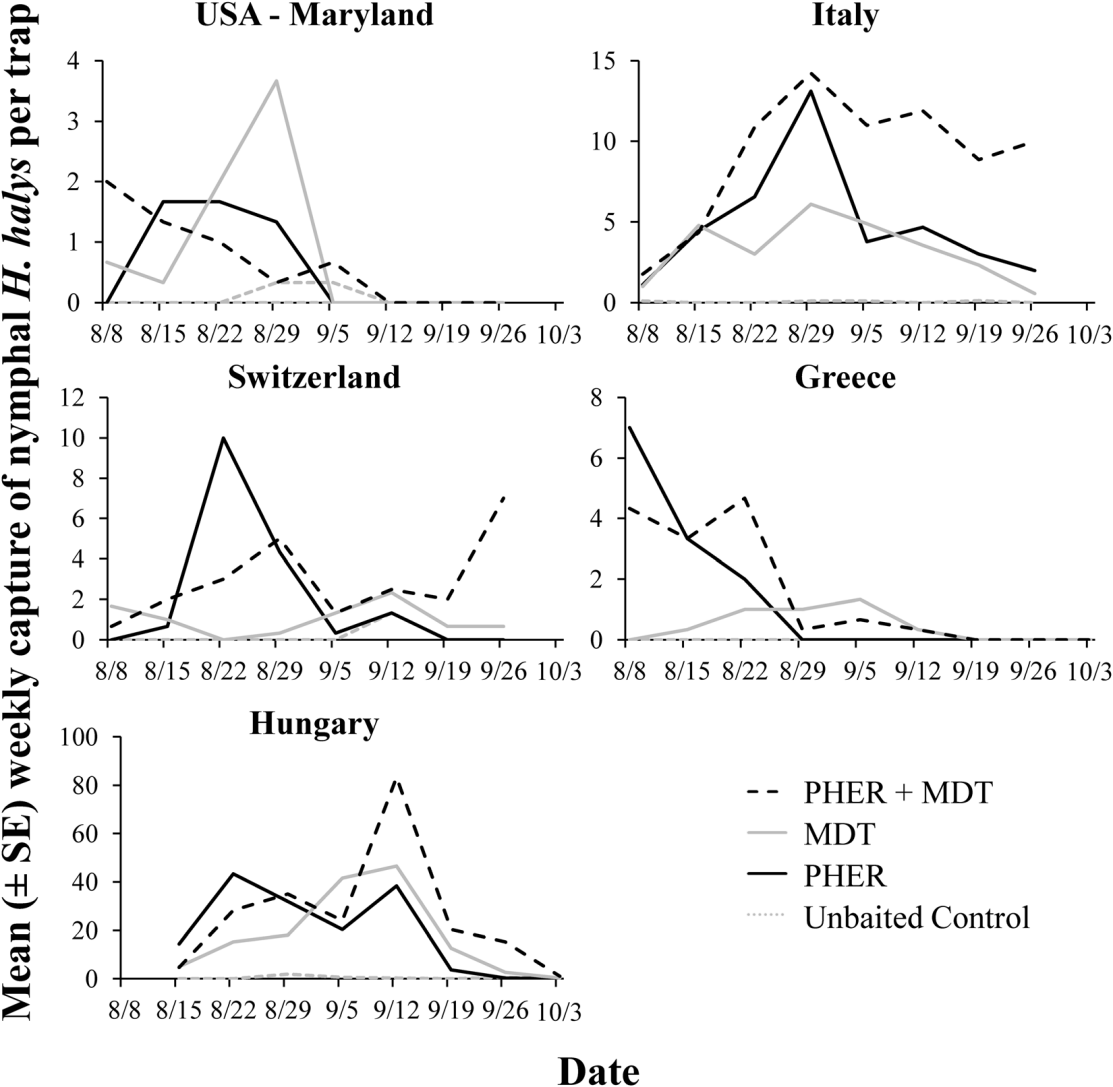
Weekly population dynamics of *H. halys* nymphs, depending on semiochemical treatment, from 8 Aug to 6 Oct 2016 in each country (Greece, Hungary, Italy, Switzerland, and USA). Abbreviations: PHER = *H. halys* aggregation pheromone, MDT = methyl (2*E*,4*E*,6*Z*)-2,4,6-decatrienoate, and CTRL = unbaited control.

In total, 50 and 2,984*H. halys* nymphs were captured in the USA (Maryland) and Europe, respectively, with 51% of the nymphs in Europe from Hungary. Similar to the adults, the semiochemical treatment significantly influenced the capture of nymphs (LMM: χ^2^ = 13.68; df = 3; *P* < 0.01; Fig. 1). In particular, there were 6–7 times more nymphs captured on traps in European countries with traps paired with MDT, PHER, or PHER + MDT compared to the unbaited controls (Fig. 1, Tukey’s HSD); unbaited traps in Maryland, USA did not capture a single nymph. The sampling country (Greece, Hungary, Italy, Switzerland, or USA) significantly influenced the captures of nymphs (χ^2^ = 30.4; df = 4; *P* = 0.0001), though this appears to be a quantitative difference in captures and not a qualitative difference in capture pattern. The sampling date significantly affected the capture of nymphs (χ^2^ = 40.9; df = 8; *P* < 0.0001), with nymphal captures peaking near the middle of the study period (Fig. 4). Unlike adults, the presence of MDT with PHER did not result in a synergistic effect on attraction of nymphs in any of the countries where sampling took place (Greece: *t* = 0.19; df = 27; *P* = 0.85; Hungary: *t* = 0.88; df = 24; *P* = 0.38; Italy: *t* = 2.19; df = 72; *P* < 0.05, but not more than twice the capture of PHER only traps; Switzerland: *t* = 0.41; df = 24; *P* = 0.68; USA: *t* = 0.25; df = 24; *P* = 0.80).

### Haplotyping Study

**Figure 5:**
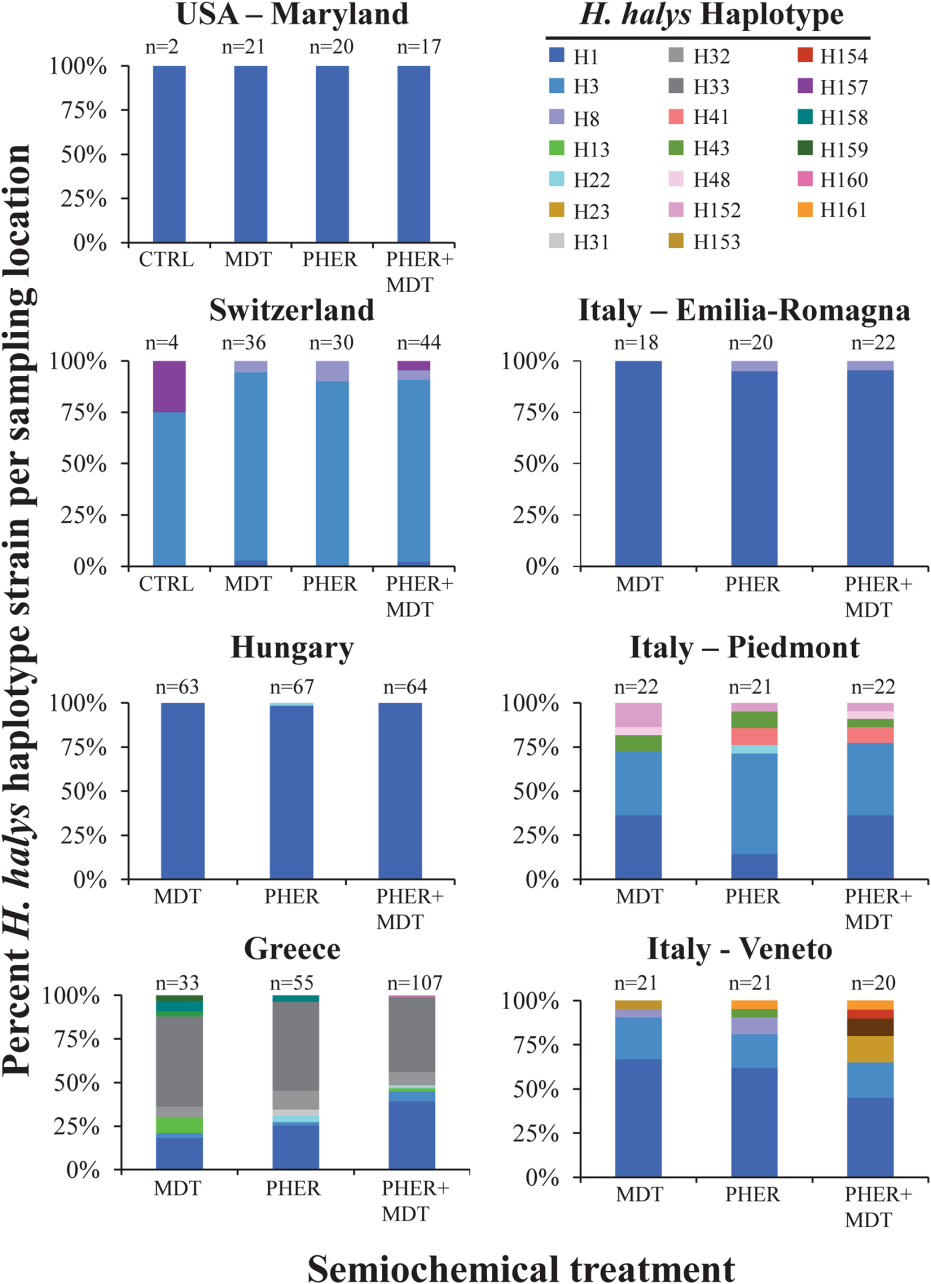
Summary of haplotype frequency of adults responding to pheromone-baited traps, and broken down by countries and regions within countries (where applicable). Sample size of analyzed adults is given above each bar, and definitions for abbreviations are as follows: MDT = methyl (2*E*,4*E*,6*Z*)-2,4,6-decatrienoate, PHER = *H. halys* aggregation pheromone. The notation for the haplotypes is in accordance with previously published literature. If a treatment bar has been omitted (e.g. the unbaited control), no or very few adults  were found in that treatment.

Sequences of the mitochondrial cytochrome oxidase I (mtCOI) gene fragment were obtained from a total of 750 specimens of *H. halys* that were collected from traps paired with no semiochemicals, MDT, PHER, or both PHER + MDT, located in the USA (Maryland) and across four European countries (Switzerland, Hungary, Greece and Italy). Among the 20 different haplotypes retrieved, five of them (H157 – H161) were detected for the first time and have not been previously described (Fig. 5). Three of those (H158, H159, H160) came from Greek specimens, while the remaining two came from Switzerland (H157) and Italy – Veneto (H161). Nucelotide sequences of each new haplotype were submitted to GenBank under the accession numbers MF120271 to MF120275. The remaining haplotypes correspond with the available sequence data from previous studies.

Veneto and Piedmont (Italy) as well as Greece were the most diverse, with the presence of eight, seven and 11 different haplotypes, respectively (Fig. 5). Conversely, remarkable haplotype homogeneity was detected in the USA (Maryland), Hungary and Emilia-Romagna in Italy, with just one, two, and two haplotypes detected, respectively. In Switzerland, there was intermediate haplotype diversity, with a presence of three previously described haplotypes and a novel one (Fig. 5).

The H1 haplotype was the only one shared among specimens from all five countries, and was found with the overall highest percentages in the USA, Hungary and Italy, regardless of semiochemical treatment (range: 14–100% of total analyzed adults in each treatment; Fig. 5). Four haplotypes were detected in more than one country, while 15 haplotypes were country-specific (Fig. 5).

The site in Maryland, USA was the only location that did not reveal any genetic diversity, with the existence of a single haplotype (H1) in all of the specimens tested. Specimens collected from traps of all the three treatments in Hungary were dominated by haplotype H1, while a single individual from a trap paired with PHER belonged to the haplotype H22. Samples from Switzerland were comprised of four different haplotypes (H1, H3, H8 and H157). Adults from traps with the combined semiochemicals belonged to all four haplotypes, whereas adults from the unbaited (control) traps belonged only to haplotypes H3 and H157. Unlike Hungary and USA, the dominant haplotype in Switzerland is H3, with equally high percentages in all of the four treatments (75–91.7%). The haplotypes H1, H3 and H8 from Switzerland have already been described from previous studies (Gariepy *et al*.^12,33^; Cesari *et al*.^8^), but haplotype H157 was found for the first time.

Specimens collected from Greece were the most diverse, displaying 11 of the 20 haplotypes detected in this study. Three of these (H158, H159, H160) were unique for Greece and have not been found anywhere else in the world so far. Each of them was captured in traps paired with attractive semiochemicals. Amongst them, the haplotypes H159 and H160 were identified in only one individual each. In addition, H13, H31, H32, and H33 haplotypes were only found in Greece, though they have been previously described (Gariepy *et al*.^33^). Among the various haplotypes, H33 was the most frequently reported, with high percentages of total analyzed adults in all treatments (range: 43–52%), while the rest of the haplotypes were detected in lower percentages (range: 0.94–39%: Fig. 5).

Italy was the second most diverse population, with 2–8 haplotypes of the 20 represented, depending on the specific Italian region. The lowest haplotype diversity was found in Emilia-Romagna (2 haplotypes), while higher diversity was found in Veneto (8 haplotypes) and Piedmont (7 haplotypes). Haplotype H1 occurs in all three regions investigated, but was the most dominant in Veneto and Emilia-Romagna, whereas the H3 haplotype predominated in Piedmont. H22, H41, H48 and H152 were unique haplotypes for Piedmont while H23, H153, H154 and H161 were unique for Veneto. The occurrence of the H161 was recorded for the first time, albeit at low frequency; it was detected in only two individuals from traps paired with either PHER, or the combined stimuli. Only the H1 and H8 haplotypes were detected in Emilia-Romagna. Only two individuals out of 60 belonged to the H8 haplotype, each captured with traps containing semiochemical stimuli (Fig. 5).

## Discussion

This is the first study to evaluate the specific haplotypes captured by baited traps in the field in Europe and compare them to those on traps in the United States. This study has demonstrated similar qualitative patterns of response to traps with lures containing PHER + MDT across six sites in four European countries compared with Maryland, USA, and is in alignment with prior work documenting the response of *H. halys* across the USA^40^. There was a synergistic effect of combining the MDT with PHER on attraction of adults in Europe and the USA, as has been described previously^39^. However, this effect was not found for attraction of nymphs, likely due to their patchy and clumped distribution in the field^46,47^. Nonetheless, for both adults and nymphs, significantly higher captures were found in traps that contained the combined stimuli in USA (Maryland) and Europe. The behavioral response of *H. halys* to traps in Europe is also similar to responses observed to traps with similar stimuli in the Republic of Korea^41^. In addition, the use of clear sticky cards as a trapping mechanism appeared to be effective in this study, further expanding the repertoire of effective trap designs^42^. This suggests that the pheromone-based tools developed in the USA have worldwide applicability, possibly including the use of traps for monitoring to inform decision-making^48^, and for  attract-and-kill^49^, though these specific tactics need to be validated in other parts of the world.

About 2–5 times more *H. halys* were found across the sites in Europe compared to the site in Maryland, USA. This may have resulted in several of the quantitative interactions in the study’s results, and is likely explained by the fact that the site in Maryland was a commercial apple orchard regularly treated with broad-spectrum insecticides to manage for *H. halys* ^50–52^, while none of the European sites were sprayed with insecticides. Short *et al*.^48^ demonstrated that when orchards are not managed with insecticides in the USA, traps with PHER + MDT yield roughly the same magnitude of adult and nymphal trap capture as we have found at unmanaged sites in Europe.

In correspondence with prior literature, we found much higher COI haplotype diversity in Europe compared to the USA^12,33^, with 20 and a single haplotype(s) found in each region, respectively. Despite extensive prior analysis of the haplotype diversity in Europe (e.g.^8,12,33,34,45^), we have documented five new haplotypes, suggesting that there is ongoing invasion and re-introduction of *H. halys* in Europe. This ongoing invasion is likely the result of human-mediated transport of overwintering adults^53^ and via the strong dispersal capacity of *H. halys* ^54^. Cesari *et al*.^34^ described 13 new haplotypes from Italy, and six of the haplotypes found in the current study match the strains they captured. By contrast, the low number of haplotypes found in the USA could be indicative of a single introduction, at least in the eastern USA, though there may have been multiple introductions in the western USA^34^ (Hoelmer *et al*., unpublished data). While consistent with prior data, the low haplotype diversity in the eastern USA may also be in part due to the fact that sampling took place in an intensively managed area with frequent insecticide applications. Future sampling for *H. halys* in unmanaged areas in the eastern USA will be able to confirm this pattern.

Importantly, there was broad-based attraction by a range of haplotypes to the semiochemical stimuli. While we did not directly assess the frequency of haplotypes in the population, prior research in Europe has done so through hand collections and other protocols that were independent of pheromone-baited traps^8,12,33,34,45^. Those data indicate that the frequency of haplotypes obtained in our study was in apparent proportion with the natural abundance of those haplotypes in the population for Italy, Switzerland, Hungary, and Greece, thus demonstrating broad-based attraction to the currently available pheromone technology regardless of specific haplotype.

There have been a variety of tools that have been important for understanding the invasion ecology of *H. halys*, including citizen science^21^, web-based tools^55^, haplotyping (e.g.^12,33^), landscape and spatial analyses^53,56^, black lights^57^, protein-marking and harmonic radar^58^, and overwintering shelters^59^, among other techniques. We have demonstrated here that we can reliably use pheromone-based technology for surveillance of *H. halys* in its introduced range, and link it with genetic data to help increase our understanding both of its chemical ecology and invasion biology. Interestingly, using the pheromone traps, we were able to pick up rare haplotypes in the environment. For example, several of the detected haplotype strains were composed of either singletons or doubletons, and for the most part, these were captured successfully on traps paired with either the *H. halys* aggregation pheromone, or combined stimuli. Ultimately, pheromone-based technology should prove useful in helping to mitigate the worldwide risks posed by *H. halys* through ongoing monitoring and management efforts, especially when used in conjunction with other IPM strategies.

## Materials and Methods

### Study Sites

There were a total of seven sampling sites spread across five countries, including Greece, Hungary, Italy, Switzerland, and the United States (Table 1). At these sites, we compared the attractiveness of the semiochemical stimuli described above when deployed with traps to ambient haplotypes present in the landscape according to country and location within country (e.g. Italy). The landscape ranged from rural to urban, with plants in the landscape that included documented *H. halys* hosts (www.stopbmsb.org; Table 1). Only one site had regular insecticide applications targeted against *H. halys* (Smithsburg, Maryland, USA: Table 1).

**Table 1:** Summary of sampling sites in Europe and the United States during the trapping study in 2016.

Country	State/Area	Town	GPS Coordinates	#Reps	Sampling Dates	Adjacent Vegetation	Landscape	Location of Traps	Insecticide Usage
USA	Maryland	Smithsburg	39.637506, 77.590428	3	10 Aug –26 Sep	*Malus pumila*	Rural	Perimeter of Orchard	Yes^1^
Italy	Piedmont	Grugliasco	45.066806, 7.590611	3	9 Aug –27 Sep	*Ulmus minor, Platanus x hybrida, Tilia cordata, Pyracantha coccinea, Populus alba, Ailanthus altissima, Acer pseudoplatanus, Prunus avium, Pyrus communis*	Suburban	Edge of wooded border/buildings	No
Italy	Veneto	Legnaro	45.345028, 11.956497	3	9 Aug –26 Sep	*Picea abies, Hybiscus spp., Acer platanoides, Cedrus libani, Ficus carica, Salix alba, Ligustrum vulgaris, Carpinus betulus, Quercus robur*	Suburban	Edge of wooded border/hedgerow	No
Italy	Emilia-Romagna	Reggio Emilia	44.691761, 10.67185	3	8 Aug –27 Sep	*Corylus avellana, Cornus sanguinea, Prunus* sp.*, Cornus mas, Acer* sp.*, Ligustrum* sp.*, Robinia pseudocacia, Morus* sp.	Suburban	Edge of wooded area	No
Hungary	Budapest	Budapest	47.3975, 19.1472	3	18 Aug –6 Oct	*Acer negundo, Ailanthus altissima, Amorpha fruticosa, Euonymus europaeus, Juglans regia, Sophora japonica, Syringa vulgaris*	Suburban	Edge of wooded border	No
Switzerland	Basel-Stadt	Basel	47.5525, 7.601667	3	8 Aug –26 Sep	*Catalpa bignonioides, Ilex aquifolium, Acer* sp.*, Rhamnus frangula, Eleocharis dulcis*	Urban	In park	No
Greece	Attica	Kifisia	38.082503, 23.81225	3	9 Aug –4 Oct	*Olea* sp.*, Quercus* sp.*, Pinus* sp*., Viburnum* sp.*, Ligustrum* sp.	Suburban	Edge of wooded border/hedgerow	No

^1^Orchard was actively managed with insecticide for *H. halys*.

### Trapping Study

The goal of the trapping study was to evaluate the population-level response of *H. halys* in Europe to semiochemical stimuli. Clear sticky cards (15.3 × 30.5 cm, STKY™ Dual Panel Adhesive Trap, Trécé, Inc., Adair, OK, USA) were hung horizontally in or near *H. halys* host trees with twist ties at a height of 1–1.5 m from the ground. Every 2 weeks, the clear sticky cards were replaced with new ones. Clear sticky cards were used as prior research has shown that they are effective for surveillance of *H. halys* in the landscape, but are also cheaper than the conventional large pyramid traps (Leskey *et al*., unpublished data). At each study site, there were a total of 3 replicate transects. Each transect was spaced at least 50 m apart. In each transect, there were one of four treatments paired with each clear sticky card: *H. halys* aggregation pheromone alone (PHER: 20 mg of murgantiol containing the two active stereoisomers; Trécé, Inc., Adair, OK, USA), MDT synergist alone (200 mg), both the *H. halys* aggregation pheromone (20 mg murgantiol) and MDT (200 mg), or an unbaited control. Within transects, each treatment was spaced at least 50 m apart to avoid trap interference. The lures have been documented to last 8 weeks, and thus did not need to be changed during the sampling interval. Traps were checked on a weekly basis for the presence of *H. halys* adults (males and females) and nymphs from 8 Aug to 6 Oct 2016. The treatments were sequentially rotated within each replicate every two weeks. At each check, all adults and nymphs were individually removed with sterilized forceps. A subset of these individuals (at least 9 stink bugs per treatment per week, or the maximum number available) were placed separately into 2.0 ml centrifuge tubes, capped, and brought back to the lab for the haplotyping procedure described below.

### Haplotyping Study

In order to assess the behavioral response of the haplotypes to the various semiochemical treatments, a subset of adults captured in the trapping study above were haplotyped. Once individuals were collected, 96% ethanol was added to each centrifuge tube, and the specimens were stored at −20 °C until used for analysis. A single leg was removed from each specimen of *H. halys* using sterilized blades and used for the DNA extraction. Genomic DNA (gDNA) was extracted by using the cetyltrimethyl ammonium bromide (CTAB) DNA isolation method as previously described^60^. The extracted DNA was used as the DNA source for the polymerase chain reaction (PCR). The primers LCO - 1490 (5′-GGTCAACAAATCATAAAGATATTGG-3′) and HCO – 2198 (5′-TAAACTTCAGGGTGACCAAAAAATCA-3′)^61^ were used to amplify a 658 bp fragment of the mitochondrial cytochrome oxidase I (COI) gene. Two microliters of the gDNA extract were used as the template in 20 μl reactions containing 0.2 mM dNTPs, 1.0 μM of each primer, 1 μl Kapa Taq DNA polymerase (Kapa Biosystems) and 1x enzyme buffer. PCRs were performed under the following conditions: one step of initial denaturation at 95 °C for 3 min; 5 cycles at 95 °C for 1 min, 45 °C for 1 min and 72 °C for 1 min; 35 cycles at 95 °C for 1 min, 50 °C for 1 min and 72 °C for 1 min and one step of final extension at 72 °C for 2 min. The amplified products were visualized on a 1.2% agarose gel containing Midori Dye, Green Staining. The PCR products were purified using the NucleoFast PCR Clean-up kit (Macherey - Nagel, Düren, Germany) according to the manufacturer’s instructions and sequenced in both directions using the primers mentioned above by Macrogen sequencing service (Macrogen Inc., Amsterdam, the Netherlands). Sequences obtained in the present study were analyzed using BioEdit v.7.0 software^62^ and were compared with the corresponding ones available in GenBank using the BLAST algorithm of NCBI^8,12,33,34^. Haplotype denotation is consistent with prior studies.

### Data Analysis

Four linear mixed models were used to analyze the trapping data from the study. In particular, either the abundance of adults or nymphs were used as a response, and males or females were considered as responses separately to evaluate any differences in responses to the semiochemicals by sex. In each case, the semiochemical treatment, sampling date, and country (Greece, Hungary, Italy, Switzerland, and USA) was used as a fixed, explanatory variable, while the field site replicate was used as a random blocking variable. Sampling week was used as a repeated measures with a first order autoregressive correlation/covariance matrix. Because the data did not conform to a normal distribution, they were log-transformed, after which assumptions were met. Wald tests for significance were performed based on a χ^2^-distribution. Upon a significant result from the model, pairwise comparisons were conducted with Tukey’s HSD. R Software was used for this and all subsequent analyses^63^, with α = 0.05.

To test whether MDT has a synergistic effect on attraction of adults and nymphs to traps with PHER in Europe and in the USA, the following procedure was used (after^42^). A t-test was used to assess whether mean captures of a given life stage in traps with PHER alone were significantly different compared to traps with the combined stimuli (PHER + MDT). If there were significant differences, and the absolute value of trap captures in traps with the combined stimuli was >2 times more than trap capture with PHER alone, then this was taken as evidence for synergism in attraction.

### Statement on Availability of Datasets

The datasets generated and analyzed during the current study are available from the corresponding author upon reasonable request. Nucleotide sequences have been deposited into NCBI GenBank as described above.

### Statement on Ethics and Informed Consent

This study did not include research on vertebrates or humans. All studies were carried out in accordance to the highest relevant ethical, scientific, and institutional guidelines in each of the authors’ countries.

## Electronic supplementary material

Supplemental Table 1
