# Supplementary material for: Attraction of Halyomorpha halys (Hemiptera: Pentatomidae) haplotypes in North America and Europe to baited traps
Source: Sci Rep. 2017 Dec 5;7:16941. doi: 10.1038/s41598-017-17233-0 (PMC5717242; doi:10.1038/s41598-017-17233-0)
Supplement: Supplementary file 1 — Supplemental Table 1 [file 41598_2017_17233_MOESM1_ESM.doc]

**Attraction of *Halyomorpha halys* (Hemiptera: Pentatomidae) haplotypes in North America and Europe to baited traps**

William R. Morrison III^1*^, Panos Milonas^2^, Despoina Evr. Kapantaidaki^2^, Michele Cesari^3^, Emanuele Di Bella^3^, Roberto Guidetti^3^, Tim Haye^4^, Lara Maistrello^3^, Silvia T. Moraglio^5^, Lucia Piemontese^3^, Alberto Pozzebon^6^, Giulia Ruocco^6^, Brent D. Short^7^, Luciana Tavella^5^, Gábor Vétek^8^, and Tracy C. Leskey^7^

^1^ USDA, Agricultural Research Service, Center for Grain and Animal Health Research, 1515 College Ave., Manhattan, KS 66502 USA

^2^ Department of Entomology and Agricultural Zoology, Benaki Phytopathological Institute, 8 St. Delta str., Kifissia, Greece

^3^ Department of Life Sciences, University of Modena and Reggio Emilia, Via G. Amendola 2, Reggio Emilia, and via Campi 213/D, Modena , Italy

^4^ CABI, Rue des Grillons 1, 2800 Delémont, Switzerland

^5^ Dipartimento di Scienze Agrarie, Forestali e Alimentari, University of Turin, Largo P. Braccini 2, 10095 Grugliasco (TO), Italy

^6^ Department of Agronomy, Food, Natural Resources, Animals and Environment – University of Padova, viale dell'Università, 16, 35020 Legnaro (PD), Italy

^7^ USDA, Agricultural Research Service, Appalachian Fruit Research Station, 2217 Wiltshire Rd., Kearneysville, WV 25430 USA

^8^ Department of Entomology, Szent István University, Villányi út 29–43, H-1118 Budapest, Hungary

^*^ Corresponding author, email: [william.morrison@ars.usda.gov](mailto:william.morrison@ars.usda.gov), tel: 785-776-2796, fax: 785-537-5584

Morrison et al. Supplementary Materials
